# Home Insulin Pump Use in Hospitalized Children With Type 1 Diabetes

**DOI:** 10.1001/jamanetworkopen.2023.54595

**Published:** 2024-02-07

**Authors:** Jodi Owens, Joshua Courter, Christine L. Schuler, Michelle Lawrence, Lindsey Hornung, Sarah Lawson

**Affiliations:** 1 Division of Endocrinology, Cincinnati Children’s Hospital Medical Center, Cincinnati, Ohio; 2 Division of Pharmacy, Cincinnati Children’s Hospital Medical Center, Cincinnati, Ohio; 3 Division of Hospital Medicine, Cincinnati Children’s Hospital Medical Center, Cincinnati, Ohio; 4 University of Cincinnati College of Medicine, Cincinnati, Ohio; 5 Division of Biostatistics & Epidemiology, Cincinnati Children’s Hospital Medical Center, Cincinnati, Ohio

## Abstract

**Importance:**

Pediatric data on inpatient home insulin pumps are absent in the literature. Understanding safety of home insulin pumps, managed by patients or caregivers, during times of illness will help diabetes technology securely move into pediatric hospitals.

**Objective:**

To examine whether insulin can be safely and accurately delivered to hospitalized children through home insulin pumps when managed by patients or caregivers.

**Design, Setting, and Participants:**

This single-center, retrospective, observational cohort study included children with insulin-dependent diabetes admitted to a tertiary children’s hospital from January 1, 2016, to December 31, 2021. In all these patients, diabetes was the primary or secondary diagnosis on admission.

**Exposure:**

Insulin delivery via home insulin pump, hospital insulin pump, or subcutaneous injection.

**Main Outcomes and Measures:**

Hyperglycemia (glucose, >250 mg/dL) and hypoglycemia (glucose, <45 mg/dL) rates (quantified as the proportion of total insulin-days), glucose variability, and diabetic ketoacidosis (DKA) recurrences were compared for hospital pumps (manual mode), home pumps (manual mode), and subcutaneous injections using bivariate tests.

**Results:**

There were 18 096 insulin-days among 2738 patients aged 0.5 to 25 years (median age, 15.8 years [IQR, 12.3-18.3 years]). Overall, 990 (5.5%) of insulin-days involved hospital insulin pumps, and 775 (4.3%) involved home pumps. A total of 155 insulin-days (15.7%) involving hospital pumps were hyperglycemic, compared with 209 (27.0%) involving home pumps and 7374 (45.2%) involving injections (*P* < .001). Moderate hypoglycemia days comprised 31 insulin-days (3.1%) involving hospital pumps compared with 35 (4.5%) involving home pumps and 830 (5.1%) involving injections (*P* = .02). Severe hypoglycemia did not differ significantly according to insulin delivery method. Two patients using injections (0.01%) developed DKA; no patients using hospital or home pumps developed DKA.

**Conclusions and Relevance:**

In this cohort study, home insulin pump use was found to be safe in a children’s hospital regarding hyperglycemia and hypoglycemia. These data support use of home insulin pumps during pediatric admissions in patients who do not require intensive care and without active DKA.

## Key Points

**Question:**

How does home insulin pump use managed by patients and caregivers compare with hospital-managed insulin pumps or insulin injections in hospitalized children with diabetes?

**Findings:**

In this cohort study of 2738 hospitalized children with insulin-dependent diabetes, hyperglycemic days were significantly decreased in those using a hospital-managed insulin pump (15.7%) and a caregiver-managed home insulin pump (27.0%) compared with those receiving insulin injections (45.2%). Moderate hypoglycemia days were significantly decreased in those using a hospital-managed insulin pump (3.1%) and a caregiver-managed home insulin pump (4.5%) compared with those receiving insulin injections (5.1%).

**Meaning:**

These findings suggest that home insulin pump use is safe among children admitted to a children’s hospital, and home insulin pump use should be considered in most non–intensive care unit admissions.

## Teaser

This cohort study of pediatric patients with insulin-dependent diabetes compares hypoglycemia and hyperglycemia rates based on type of use of insulin pumps.

## Introduction

Patient-owned insulin pumps are becoming the standard of care for diabetes in the US.^1,2^ Insulin pumps allow more accurate insulin dosing and have been associated with cost savings, estimated at $5000 to $6000 per person annually.^1,2^ From 2000 to 2010, the use of home insulin pumps by patients in the US increased by a factor of 5.^2^ Among children, estimates suggest that approximately 40% to 60% of those with type 1 diabetes use an insulin pump, and popularity is increasing.^2^ Most pediatric patients use an insulin pump or other form of technology within 6 months of diagnosis.^3^ Children who use insulin pumps benefit from improved quality of life and better glycemic control, including a reduction in glycated hemoglobin.^4^

Medical societies, including the Diabetes Science Technology Society, the Endocrine Society, and the American College of Endocrinology, have endorsed continued use of home insulin pumps during inpatient stays, if support is available and hospital policies are in place to promote safe pump use.^5,6,7,8^ Adult studies have provided guidance to help clinicians determine which patients are ideal candidates to continue home insulin pumps during hospitalization; however, decisions must continue to be made on a case-by-case basis and within the context of institutional policies.^9,10,11,12^ Hospitals nationwide continue to struggle with how involved hospital staff should be in managing home insulin pumps, especially because these devices deliver a high-risk medication (ie, insulin).

Adult safety data on home insulin pumps managed by patients during hospitalization concluded that blood glucose control is comparable to hospital-managed methods of insulin delivery. Specifically, hypoglycemic and hyperglycemic events occurred at a similar or lower rate between the 2 groups.^9,13,14,15,16^ Currently, no safety data are available pertaining to the use of home insulin pumps in children and adolescents during hospital admissions. Thus, children’s hospitals are left to extrapolate from adult safety data when determining whether to allow patients or caregivers to use home pumps during times of illness. Directly extrapolating learnings from adult studies is difficult, however. Children often require a caregiver, or multiple caregivers, to manage a home insulin pump, which could increase the complexity of in-hospital use of home pumps, among other factors. Lack of pediatric safety data are, therefore, a notable gap in the literature and must be addressed. Clinicians and institutions will continue to encounter pediatric patients who use home insulin pumps, and families are likely to prefer to continue use of their home pump during hospital stays.

The objective of this study was to evaluate inpatient home insulin pump use during pediatric hospitalizations in non–intensive care units. Specifically, we sought to compare hypoglycemia and hyperglycemia rates based on use of (1) hospital-owned and -managed insulin pumps, (2) home insulin pumps managed by a patient or caregiver, and (3) hospital-managed subcutaneous insulin injections.

## Methods

### Study Setting and Design

This retrospective, observational cohort study included children admitted to the hospital for any indication (other than diabetic ketoacidosis [DKA]) with insulin-dependent diabetes to a pediatric inpatient hospital unit. The study was conducted in a large, tertiary children’s hospital with 670 inpatient beds and approximately 1.2 million inpatient encounters yearly. The patient’s primary care practitioner (physician or nurse practitioner) places insulin orders in the electronic health record. Endocrinologists may be the primary care practitioner or serve in a consultative role. Approximately 10 400 doses of rapid-acting insulin were administered annually to inpatients at the time of the study. The institutional review board at Cincinnati Children’s Hospital Medical Center approved the study and provided a waiver of informed consent. This study followed the Strengthening the Reporting of Observational Studies in Epidemiology (STROBE) reporting guideline.^17^

All patients were identified retrospectively. The only inclusion criterion was a diagnosis of insulin-dependent diabetes as the primary or secondary diagnosis. Children who had undergone total pancreatectomy islet cell autotransplantation were excluded due to strict, extreme glucose control needs. Patients requiring intensive care were also excluded based on established adult criteria (Box).^9^ All patients were admitted to a non–intensive care medical, surgical, or psychiatric unit from January 1, 2016, to December 31, 2021. Data from patients admitted with DKA were included once DKA resolved (defined as a bicarbonate level >17 mEq/L [to convert to millimoles per liter, multiply by 1] and discontinuation of an insulin drip). Hypoglycemia and hyperglycemia rates were compared among patients using (1) hospital insulin pumps (in manual mode) managed by hospital staff, (2) home insulin pumps (in manual mode) managed by the patient or caregiver, and (3) subcutaneous insulin injections managed by hospital staff. Rates of recurrent DKA after any initial resolved episodes were also calculated. Sociodemographic information was purposely not collected because the level of diabetes education for patients or caregivers was not a requirement for study participation, proficiency of pump use cannot be directly extrapolated from social or demographic factors, and sociodemographic factors do not alter the function of an insulin pump.

Box. **Patient Findings That Led to Contraindications to Using a Patient- or Caregiver-Managed Home Insulin Pump in the Hospital^a^**

- Impaired level of consciousness if the was patient 18 years or older or caregiver if the patient was younger than 18 years
- Patient or caregiver inability to correctly demonstrate appropriate pump settings
- Critical illness requiring intensive care
- Psychiatric illness as the reason for admission or patient admitted to inpatient psychiatric unit
- Refusal or unwillingness to participate in self-care
- Lack of pump supplies
- Patient at risk for suicide
- Health care decision by primary admitting team or endocrine consult team

^a^
Box recreated from Umpierrez and Klonoff.^9^ Diabetes Technology Update: Use of Insulin Pumps and Continuous Glucose Monitoring in the Hospital, June 23, 2018. Copyright and all rights reserved. Material from this publication has been used with the permission of American Diabetes Association.

#### Hyperglycemia and Hypoglycemia

Blood glucose values were obtained via point-of-care testing or venous blood testing. Glucose measurements obtained through home continuous glucose monitors were verified via a point-of-care glucose or venous glucose test. Hyperglycemia was defined as a glucose level greater than 250 mg/dL (to convert to millimoles per liter, multiply by 0.0555). Hypoglycemia was defined as moderate (glucose, 45-59 mg/dL) or severe (glucose, <45 mg/dL). We quantified hyperglycemia and hypoglycemia in days to avoid overrepresenting events that required frequent blood glucose testing (ie, during times of glucose correction immediately after an intervention was given). An insulin-day was defined as the number of days patients received any insulin dose(s). For example, if 1 patient is receiving a unit for 5 days or if 5 patients are receiving a unit for 1 day, each unit would have had 5 insulin-days. A hospital day was categorized as a hyperglycemic-day if a patient had 1 or more glucose levels that met the definition of hyperglycemia in that calendar day or a hypoglycemic-day if a patient had 1 or more glucose levels that met the respective definitions for moderate or severe hypoglycemia. Rates of hyperglycemia were calculated by taking the number of hyperglycemic days divided by the total number of insulin-days for each respective insulin delivery method. Rates of hypoglycemia were calculated using the same approach. If a patient came in with DKA, hyperglycemia, or moderate or severe hypoglycemia, data extraction began on day 2 of admission to eliminate chaotic blood glucose trends often seen on admission.

For context, insulin use at our institution for medical and surgical patients occurs most frequently in the diabetes unit (>11 000 insulin-days per year), followed by the pulmonary unit (700-800 insulin-days per year) and oncology units (500-600 insulin-days per year). General medical or surgical units (250 insulin-days per year) and off-site psychiatry units (70 insulin-days per year) provide insulin relatively infrequently.

### Study Groups

#### Hospital-Supplied Insulin Pumps

Hospital insulin pumps were limited to patients in the diabetes unit at the discretion of the primary admitting team and/or the endocrinology team and were kept in manual mode, meaning insulin doses were not calculated by the pump automatically. Nursing staff are trained in hospital pump use and are responsible for administering all insulin basal and bolus doses through the hospital insulin pumps. Hospital insulin pumps undergo routine and rigorous safety checks completed by medical equipment specialists and warranties are maintained.

#### Home Insulin Pumps

Home pumps were permitted in all inpatient units at the discretion of the medical team. Home insulin pumps were managed by patients or caregivers who demonstrated basic pump competency to the on-call diabetes team. All pumps were required to operate in manual mode. Hospital personnel did not inspect the pumps. Patients were permitted to use their home pump to deliver insulin if they were 18 years or older and able to perform their own insulin care independently or if a caregiver with proficiency in pump management was present. Patients were required to follow standard insulin dosing guidelines, per standards set by the American Diabetes Association.^18^ All hospital nursing staff received general training on diabetes management. Nurses received no training on any type of home insulin pump. All nursing staff received instruction on documenting patient- or caregiver-reported insulin dosing for carbohydrate coverage and hyperglycemia correction, as well as basal insulin delivery rates. Bedside nurses confirmed and recorded in the electronic health record the reported insulin dose administered by the patient or caregiver.

#### Subcutaneous Insulin Injections

Patients who did not receive insulin through an insulin pump received insulin via subcutaneous injections. Additionally, if for any reason a home or hospital pump was not available or could not be safely used, insulin was converted to injections. Injections were given via standard hospital protocol.

### Statistical Analysis

Data were analyzed using SAS software, version 9.4 (SAS Institute Inc). Due to skewed distributions, age data were summarized as median (IQR). The mean (SD) for blood glucose was calculated for each group to assess glucose control and variability. Categorical data were summarized as numbers (percentages). For continuous data, Kruskal-Wallis tests were used for comparisons between groups. The χ^2^ and Fisher exact tests were used, as appropriate, for group comparisons of categorical data. Generalized linear models were used to assess blood glucose means (SDs) between groups for the first 20 insulin-days (group and time were included in the model). False discovery rate adjustments were used to adjust for multiple testing when looking at subanalyses for hospital compared with home pumps. All statistical tests performed were 2-sided. *P* < .05 was considered statistically significant.

## Results

### Patient Characteristics and Insulin-Days

There were 18 096 insulin-days among 2738 patients aged 0.5 to 25 years, with the median patient age being 15.8 years (IQR, 12.3-18.3 years) (Table). There were 990 insulin-days (5.5%) involving hospital-issued insulin pumps, 775 insulin-days (4.3%) involving patient- or caregiver-managed home insulin pumps, and 16 331 insulin-days (90.2%) involving subcutaneous insulin injections (Table).

**Table.** Adverse Events Occurring During All Routes of Insulin Administration^a^

Adverse event	Home insulin pump use (n = 775)	Hospital insulin pump use (n = 990)	Subcutaneous insulin injection use (n = 16 331)	*P* value
Hyperglycemic-day (glucose >250 mg/dL)	209 (27.0)	155 (15.7)	7374 (45.2)	<.001
Moderate hypoglycemic-day (glucose 45-59 mg/dL)	35 (4.5)	31 (3.1)	830 (5.1)	.02
Severe hypoglycemic-day (glucose <45 mg/dL)	12 (1.5)	8 (0.8)	187 (1.1)	.35
Insulin drip during visit^b^	510 (65.8)	725 (73.2)	3476 (21.3)	<.001
DKA this day^c^	0	0	2 (0.01)	>.99
Age on insulin-day, median (IQR) [range], y	15.0 (10.9-17.3) [0.5-24.8]	14.2 (10.6-16.8) [3.1-24.9]	15.9 (12.4-18.3) [0.5-25.0]	<.001

Abbreviation: DKA, diabetic ketoacidosis.

SI conversion factor: To convert glucose to millimoles per liter, multiply by 0.0555.

^a^ Data are presented as number (percentage) of patients unless otherwise indicated.

^b^ Patients who required an insulin drip on admission due to presenting with DKA.

^c^ Patients who required an insulin drip 48 hours after admission representing those who developed DKA during the admission but not at presentation.

### Hyperglycemia and Hypoglycemia

A total of 155 (15.7%) of all-insulin days involving hospital pumps qualified as hyperglycemic-days compared with 209 insulin-days (27.0%) involving home pumps and 7374 insulin-days (45.2%) involving subcutaneous injections (*P* < .001) (Table). Hypoglycemia data showed at least 1 moderate hypoglycemic-day occurring in 31 patients (3.1%) receiving insulin via hospital pumps, 35 (4.5%) receiving insulin via home pumps, and 830 (5.1%) receiving insulin via subcutaneous injections (*P* = .02) (Table). At least 1 severe hypoglycemic-day occurred in 8 patients (0.8%) receiving insulin via hospital pumps, 12 (1.5%) receiving insulin via home pumps, and 187 (1.1%) receiving insulin via subcutaneous injections (*P* = .35) (Table).

### DKA Recurrence

During hospitalizations, no patients using a hospital or home insulin pump developed DKA, including patients who were using an insulin pump after DKA resolution and those admitted to the hospital with an insulin pump with a nondiabetes admission code. We found that 2 patients (0.01%) developed DKA during their admission while managed with subcutaneous injection therapy (Table).

### Glucose Control and Variability

Mean blood glucose and variability appeared similar among patients managed with hospital and home insulin pumps for the first 20 insulin-days. Patients using home (70%) and hospital (75%) insulin pumps had mean blood glucoses levels within the desired glucose range (80-130 mg/dL) for significantly more days than patients managed with injections (0%) (*P* < .001). Mean blood glucose over time (F = 0.22, *P* = .85) and the proportion of time within desired blood glucose range (75% in hospital vs 70% in home pumps, *P* > .99) did not significantly differ between hospital and home pumps. Among patients managed with subcutaneous injections, the mean blood glucose was consistently above the desired range, and there was significantly greater glucose variability (F = 40.55, *P* < .001) with compared with those using home and hospital pumps (Figure).

**Figure.**
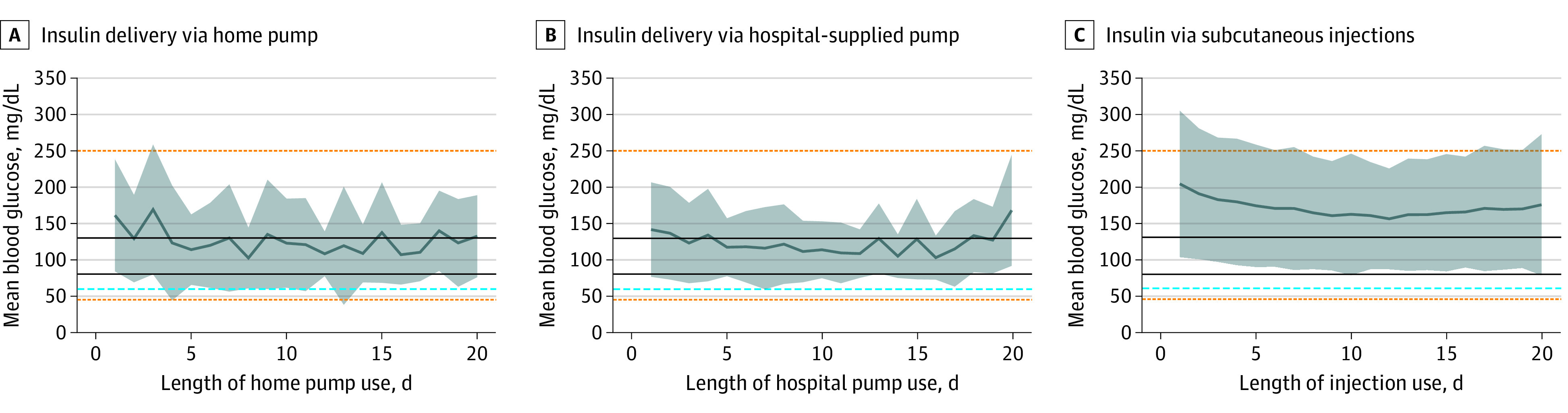
Mean Blood Glucose Concentrations for the First 20 Insulin-Days per Encounter Shading indicates SDs; solid black lines, the desired range of 80 to 130 mg/dL; orange dotted lines, hyperglycemia at 250 mg/dL and severe hypoglycemia at 45 mg/dL; and blue dashed line, 59 mg/dL for moderate hypoglycemia. To convert glucose to millimoles per liter, multiply by 0.0555.

## Discussion

In this cohort study of hospitalized children, we demonstrated the relative safety of continuing home insulin pumps in children during times of hospitalization compared with the use of medical staff–managed hospital insulin pumps and subcutaneous injections. The safety profile included assessments of hyperglycemia, hypoglycemia, and glucose variability. Our findings demonstrate that use of a home insulin pump in children during inpatient admissions is safe when done outside intensive care units in patients who do not have active DKA. Additionally, the use of a home or hospital pump reduced hypoglycemia and hyperglycemia in children when compared with children receiving insulin via subcutaneous injection. Mean blood glucose and time within the desired range were not significantly different between children using hospital vs home insulin pumps and were superior to the findings in children using injections.

During the 6-year study period, no patient in this study sample developed DKA when using a home insulin pump during an admission. Diabetic ketoacidosis developed in only 2 children receiving subcutaneous injections, presumably due to the improved glucose control seen when a home or hospital pump was used. Not only were patients within the desired blood glucose range longer when using a pump (hospital or home) during their admission, but they also achieved blood glucose control faster than those receiving injections. Severe hypoglycemia rates in patients using hospital pumps, home pumps, and subcutaneous injections were similar. Collectively, our results show that the use of insulin pumps is, in many ways, more accurate with blood glucose control than subcutaneous injections, and safety is not sacrificed when patients or caregivers use home pumps during pediatric non–intensive care unit admissions.

Each hospital and health system must make its own determinations about the risks and benefits of permitting home insulin pump use. Understandably, hospitals may hesitate to rely on patients and caregivers to deliver insulin, which is known to be a high-risk drug. Lack of control over the type of home device being used and lack of staff familiarity with the many types of home insulin pumps may also give policymakers pause. However, these factors must be balanced with patients’ or caregivers’ desires to continue using home equipment and hopes to avoid unnecessary sticks for insulin injections. Home pump use is now standard practice at our institution, with exceptions based on clinical judgment and the contraindications previously published for adults (Box).^9^ Our data indicate that the risks of relying on personal devices and dependence on caregivers and patients to use insulin pumps correctly are outweighed by the benefits of using home pumps. Home pump use minimizes the number of injections patients receive, engages the patient or caregiver in care, allows for continuity in treatment within and outside the hospital, and provides an opportunity to refine pump settings. In-hospital use of home insulin pumps is also supported by The Joint Commission with appropriate safety measures.^19^

Guidelines from multiple medical societies indicate that support must be available to facilitate safe use of home insulin pumps during hospital admissions. *Support* is a broad term and could relate to availability of supplies, technological support, or other factors. Adequate support is even more difficult to define; hospitals may have variable comfort levels, and adequate support for one hospital may not be adequate for another. Although this study was not designed to detect and characterize specific issues that may have required support during hospitalization, such as equipment malfunction, our study was conducted in a clinical setting with a broad population of medical, surgical, and psychiatric patients, where most insulin-days did not occur in a diabetes service or unit and patients and caregivers were responsible for providing all supplies. Nonetheless, the safety profile was reassuring regarding hyperglycemia and hypoglycemia. Consequently, our findings are likely broadly applicable to centers that admit insulin-dependent children to non–intensive care settings with a variety of diagnoses.

Apart from providing and documenting appropriate insulin doses and ensuring home pumps are safe in the inpatient setting, other factors and strategies for caring for hospitalized children using home insulin pumps should be considered. All pumps, regardless of model, require inspection of the pump site for any signs of infection and replacement of the pump catheter at the appropriate interval. Nurses and other medical professionals may be required to monitor pump catheter sites. Other team members, such as pharmacists and diabetes educators, may provide individual aspects of care. Team members may have evolving roles in caring for hospitalized children using home insulin pumps if more children begin to use home pumps. Hospitals may require some staff to obtain additional pump expertise as well.^20^

### Limitations

This study has limitations. First, our institution had already implemented many strategies to ensure insulin safety; it is possible that prior safety work enhanced overall insulin safety and awareness of insulin as a high-risk drug, thereby improving hyperglycemia and hypoglycemia rates. Second, this study was not designed to evaluate hyperglycemia and hypoglycemia specifically during transitions off pumps onto insulin injections or to discern the factors that contribute to stopping inpatient use of home insulin pumps. Third, this study sample did not include patients admitted to the intensive care unit. Consequently, our findings are not generalizable to patients in intensive care unit settings and patients with active DKA.

## Conclusions

This cohort study found that home insulin pump use was safe among children admitted to non–intensive care units at a children’s hospital with regard to hypoglycemia and hyperglycemia. Pediatric institutions should consider adult safety data, existing clinical guidelines that support inpatient use of home pumps, our findings, and patient and family preferences when establishing internal policies on home pump use during pediatric admissions.

## Notes

Supplement.
Data Sharing Statement
