# Supplementary material for: Home Insulin Pump Use in Hospitalized Children With Type 1 Diabetes
Source: JAMA Netw Open. 2024 Feb 7;7(2):e2354595. doi: 10.1001/jamanetworkopen.2023.54595 (PMC10851090; doi:10.1001/jamanetworkopen.2023.54595)
Supplement: Supplement. — Data Sharing Statement [file jamanetwopen-e2354595-s001.pdf]

Data Sharing Statement

Owens. Home Insulin Pump Use in Hospitalized Children With Type 1 Diabetes. *JAMA Netw Open*. Published February 07, 2024. doi:10.1001/jamanetworkopen.2023.54595

Data
Data available: No
